# A Novel Technique for Improving Cyclic Behavior of Steel Connections Equipped with Smart Memory Alloys

**DOI:** 10.3390/ma17133226

**Published:** 2024-07-01

**Authors:** Ali S. Alqarni, Mohammad J. Alshannag, Mahmoud M. Higazey

**Affiliations:** Department of Civil Engineering, College of Engineering, King Saud University, Riyadh 11421, Saudi Arabia; mjshanag@ksu.edu.sa (M.J.A.); mhigazey@gmail.com (M.M.H.)

**Keywords:** shape memory alloy, self-centering, steel connection, hollow column, residual drift, ABAQUS

## Abstract

Residual drifts are an important measure of post-earthquake functionality in bridges and buildings, and can determine whether the structure remains fit for its intended purpose or not. This study aims at investigating numerically, through finite element (FE) analysis in ABAQUS, the cyclic response of exterior steel I beam-hollow column connection using welded shape memory alloys (SMA) bolts and seat angles. This is followed by validating the numerical model using an accredited experimental data available in the literature through different techniques, (1) SMA bolts, (2) SMA angles, (3) SMA bolts and angles. The parameters investigated included: SMA type, SMA angle thickness, SMA bolt diameter, SMA angle stiffener and SMA angle direction. The cyclic performance of the steel connection was enhanced further by varying the bolt diameter, plate thickness, angle type and direction. The results revealed that the connections equipped with a combination of SMA plates and SMA angles reduced the residual drift by up to 94%, and doubled the self-centering capability compared to conventional steel connections. Moreover, the parametric analysis showed that Fe-based SMA members could be a good alternative to NiTi based SMA members for improving the self-centering capability and reducing the residual drifts of conventional steel connections.

## 1. Introduction

The devastating earthquakes that hit the mediterranean region recently, urged the need for developing smart systems and materials capable of minimizing the damage that could lead to human losses, and substantial maintenance costs. Smart systems are defined as systems that can mechanically adjust their structural response under various loading conditions. Smart materials are the basic elements [1] that can be integrated into the smart systems to provide the actuation mechanism and the self-stressing component. The latest advances in materials science and cutting-edge processing technologies have led to discovery of shape memory alloys (SMAs) that have the capability to recover their pristine shape after a significant deformation of about 12% strain [2,3]. The superiority in the mechanical properties of SMA over steel is attributed to their ability to recover high deformations (self-centering) when subjected to reversed cycling loading simulating earthquake excitations. Such behavior makes the SMAs unique materials and potential candidates for improving the serviceability of structures subjected to severe earthquakes. Different types of SMA based materials have been developed lately, including NiTi (nickel–titanium) alloy, copper (Cu), niobium (Nb), and iron (Fe) [4,5,6,7]. Cu-based SMAs are relatively inexpensive materials with a recoverable strain limited to 2–4% [8]. Another potential low-cost SMA type is Fe-based that have a limited use in industry sector, because of manufacturing constraints [9]. NiTi SMAs are commonly used nowadays in civil engineering structures because they can display excellent superelastic properties, high damping and actuation capacities [10], and superior self-centering capability. However, due to the high cost of SMAs, their optimized applications are limited to critical regions, including beam-column connections [11], bridges, frequency controllers [12,13], damper [14,15,16,17], bracing systems [18,19], and vibration isolation systems [20,21,22].

Shape memory effect (SME) and superelasticity (SE) are two distinct properties of SMA compared to other materials. SME allows the material to return to its original shape after deformation when heated, while superelasticity enables the material to recover from large strains without causing permanent deformation under loading/unloading process [23]. The behavior of SMA is highly influenced by phase transformation temperatures. The SME and SE in SMA primarily arise from changes in their crystal microstructures. SMA exhibits three crystal structures: twinned martensite, detwinned martensite, and austenite. Macroscopically, SMA behavior under thermal and/or mechanical loading or unloading can be categorized into two phases: martensite, which is a weaker phase and stable at lower temperatures, and austenite, a stronger phase and stable at higher temperatures. Further details about the behavior of SMAs under the effects of various temperatures are discussed by Alshannag et al. [6]. Superelasticity, or sometimes referred to pseudoelasticity, is measured via cyclic tensile testing per ASTM F2516 [24]. The SME is triggered by a thermal transformation between the high-temperature austenite phase and the low-temperature martensite phase. The specific temperatures at which these transformations occur, and the shape of the hysteresis loop depend on the composition of the alloy and the processing method [25]. Welding of SMAs is a key challenge in practical infrastructure applications due to their unique thermomechanical properties. Researchers have made significant progress in developing welding and joining techniques for SMA materials in recent years. The literature highlights several promising approaches for both Fe-based and NiTi SMAs. For instance, Lin et al. [26] demonstrated that Fe-based SMAs can be successfully laser welded. However, this process requires subsequent annealing treatment. Zhou et al. [27] reported on the use of crossflow laser welding to join Fe-based SMA components and found that the tensile strength of the welded joints could achieve 94% of the base material strength with optimal welding parameters. Zhao et al. [28] improved the mechanical properties of laser-welded NiTi alloy samples by incorporating additives into the welding process, resulting in higher tensile strength and toughness. Similarly, Santos et al. [29] showed promising results using laser brazing to join NiTi to stainless steel. Alam et al. [30] pointed out that Fe-Mn-Si-based SMAs may offer advantages over NiTi in terms of weldability due to their superior mechanical and welding characteristics. Generally, the advancements in welding and joining techniques have the potential to enable broader and more reliable use of SMA in engineering applications.

Steel structures continue to gain more popularity in active seismic regions due to their superior mechanical performance, high energy absorption and dissipation capacities, and ease of fabrication. The construction industry sector has witnessed a rapid increase in using steel sections of I and H shapes for beams and columns during the recent years. Additionally, the steel columns of hollow cross sections have gained more acceptance among the structural engineers and industry sector nowadays. This is because of their greater load carrying capacity, better fire resistance, lesser maintenance cost, faster manufacturing and fabrication of the section, and ease of filling it with concrete and cement based composites. However, I beam-hollow column connections require rigorous fabrication and detailing, which increases the overall cost of such a system and limits its use in practical applications. Despite these fabrication difficulties, various approaches were proposed in the literature to enhance their performance such as blind bolts [31], welding the beam to column [32], and using welded bolts [33]. Nevertheless, steel moment resisting frames experience severe damage under strong earthquakes if critical regions are not adequately designed and detailed. The permanent deformations, generally referred to as residual drifts are caused due to yielding of steel in the beam-column joint. This results in high post-earthquake maintenance costs or may lead to the probability of a partially or fully demolition of a structure [34,35]. Furthermore, it has been reported that the steel structures with residual drifts caused by seismic events more than 0.005 rad, could be demolished due to economic considerations [36]. Various studies were carried out to investigate the use of the SMA elements for the purpose of reducing the residual displacements and improving the self-centering capacity of steel beam-column connections. Moradi and Alam [37] studied numerically the seismic performance of welded steel I-beam to hollow column connections with FeMnAlNi SMA plates placed in the plastic hinge region of the beam. The results showed that the SMA plates were very effective in reducing the residual deformation by 23–90% compared to the control specimen. Chowdhury et al. [38] investigated numerically the influence of SMA strands on the cyclic performance of the steel I-beam to I-column connections and showed that the specimens equipped with CuAlMn and FeNCATB SMA strands performed well in terms of stiffness and self-centering ability. Furthermore, Chowdhury et al. [39] proposed using end plates with SMA bolts for a further enhancement in the self-centering capability of the steel I-beam to I-column connections. Torabipour et al. [40] studied numerically the use of bolted SMA T-stub for steel H-beam to H-column connections and indicated that using stiffeners for T-stub increased the energy dissipation by 32%. Wang et al. [41] experimentally tested NiTi SMA angle and concluded that the SMA angle can exhibit satisfactory flag-shaped hysteresis loops under reversed cyclic loading.

The literature reviewed indicated that scarce information exists on the cyclic behavior of steel I beam-hollow column connections equipped with smart alloys. The current investigation proposes an innovative technique for enhancing the cyclic performance of conventionally designed steel beam-column connections without compromising their moment capacity. This technique involves using different types of smart memory alloys, in the form of welded SMA angles and bolts for enhancing the performance of the connections. Properties investigated included hysteretic response, moment capacity, self-centering capability, residual deformations, energy dissipation, flexural stiffness and stiffness degradation. The structural response of the steel connection started by a numerical FE modelling using ABAQUS 2020 software and validated based on an accredited experimental data published by Serrano-López et al. [33]. The cyclic performance of the steel connection was optimized further through a parametric study using the FE model developed. Four approaches were considered to study the effect of the SMA on behavior of the connections compared to conventional steel connections using: (a) steel bolts and steel angles (b) SMA bolts and steel angles, (c) steel bolts and SMA angles, (d) SMA bolts and SMA angles. Due to the lower costs and better weldability of Fe-based SMAs compared to NiTi [26,27,28,29,30], each approach was tested using both alloys NiTi and FeMnAlNi. The numerical study was further expanded to include the effect of some parameters on the self-centering behavior of the I beam-hollow column SMA connections: (1) the effect of the SMA materials (NiTi vs. FeMnAlNi), (2) the effect of thickness of the angles (8 mm vs. 10 mm vs. 12 mm), (3) the effect of angle direction (long vs. short leg), (4) the effect of using stiffener (with vs. without stiffeners), and (5) the effect of the bolt diameters (20 mm vs. 25 mm vs. 30 mm). The results of this study provide a novel technique for utilizing a combination of smart angles and bolts for enhancing the seismic performance of conventional steel frames under reversed cyclic loading. Moreover, the parametric study sheds the light on the most influential parameters affecting the self-centering capability of steel connections equipped with smart alloy.

## 2. Experimental Reference

**Figure 1:**
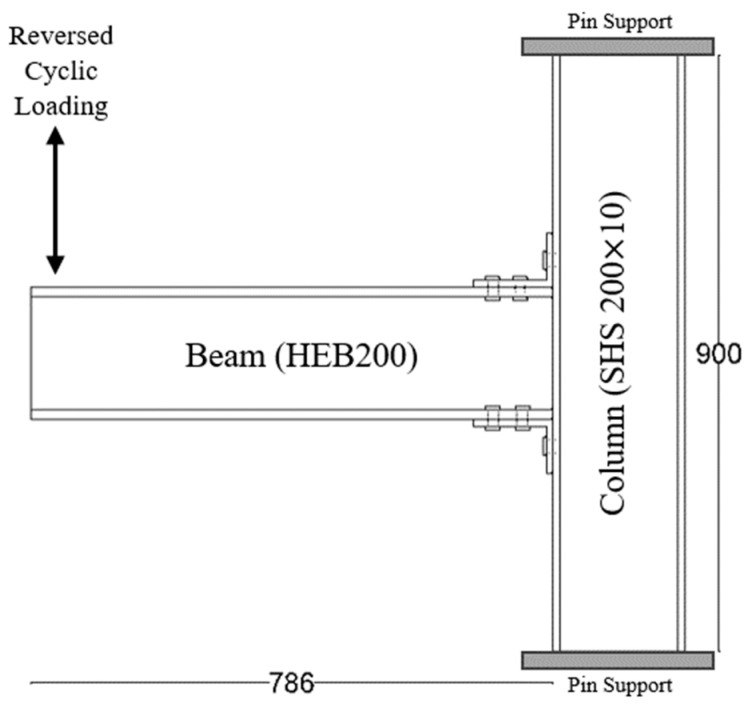
Sketch of the column restraining system, elevation, and plan views (all dimensions are in mm).

**Figure 2:**
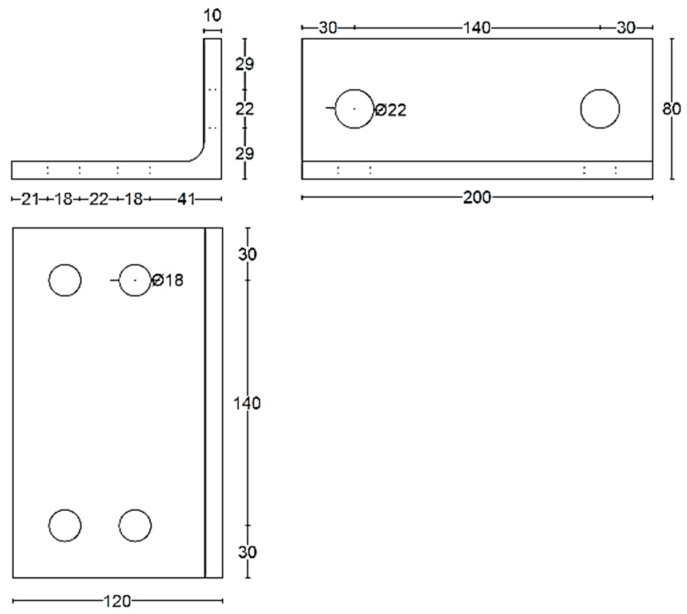
Hole positions and dimensions in the angle (all dimensions are in mm).

**Figure 3:**
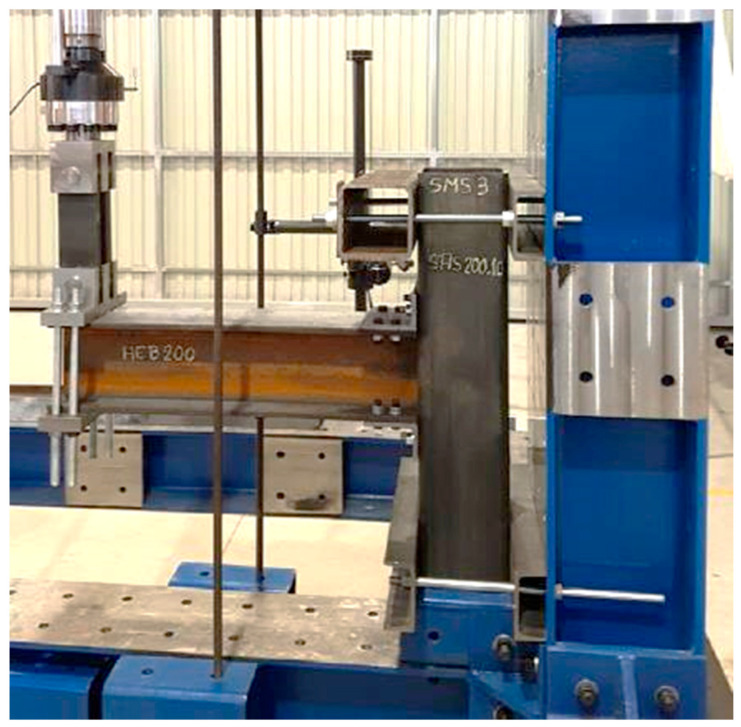
Test set-up of the beam-column connection. Reprinted from [33], with permission from Elsevier.

**Figure 4:**
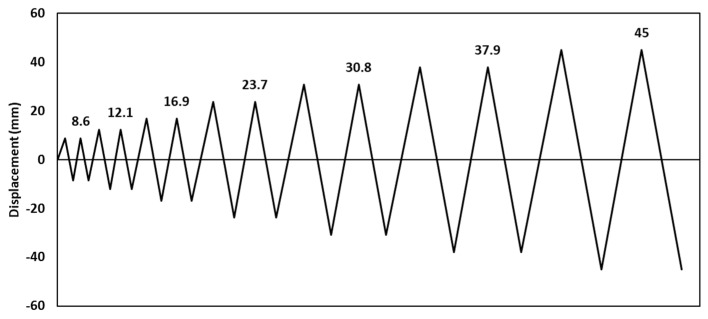
Cyclic loading history according to FEMA-461 [43]. Reprinted from [33], with permission from Elsevier.

The experimental investigation performed by Serrano-López et al. [33] was used as a main accredited reference for the verification of the three-dimensional finite element (FE) models developed in this study using ABAQUS [42]. The experimentally tested specimens consisted of a steel I beam to square hollow section column joints connected by steel angles. The flange of the beam was connected to the column using bolted top seat angles, as shown in Figure 1. The sections of the column, beam, and angles were SHS 200 × 10, HEB 200 and L 120 × 80 ×10, respectively. Two threaded welded studs were used to connect the short leg of the angle to the column, whereas the four ordinary bolts were used to connect the long leg of the angles with the beam. M20 Grade 4.8 was used for the welded bolts connected to the column whereas M16 Grade 10.9 was used for ordinary bolts connected to the beams. The positions and dimensions of the holes placed on the angles are presented in Figure 2. The column was fixed to the frame using steel rods at the top and bottom ends of the column to prevent any horizontal or vertical displacement during the test, as shown in Figure 3. A quasi-static cyclic load was performed on the tested specimen according to FEMA-461 [43], as shown in Figure 4.

## 3. Finite Element Modelling

A three-dimensional finite element model was developed using ABAQUS software [42] to simulate the cyclic performance of I-beam-hollow column connection. The following subsections describe the employed elements, interaction, boundary conditions, and material models.

### 3.1. Elements, Interactions, and Boundary Conditions

**Figure 5:**
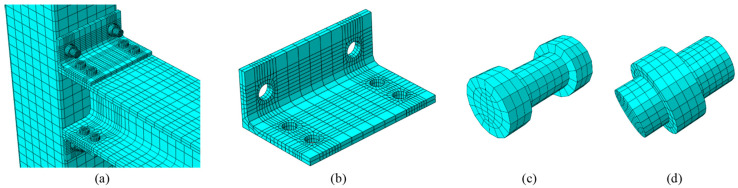
Meshed elements (**a**) full specimen, (**b**) angle, (**c**) ordinary bolt, and (**d**) welded bolt.

The 8-node solid (C3D8R) elements were employed for modelling all the parts: column, beam, angle, and bolts. Since no welding failure was observed in the test specimens, a tie contact interface was used to simulate the welding contact between the welded bolts and the column. This type of contact was successfully adopted by many researchers [44,45,46,47,48]. A general contact type in ABAQUS was used to simulate the interaction between the connected elements. Figure 5 presents the discretized mesh of the FE elements (column, beam, angle, and bolts), with element mesh sizes varying from 30 to 4 mm. A mesh sensitivity analysis was initially performed to determine the optimum mesh size for all elements of the beam-column connection that would produce accurate results with less computational time. The mesh size for the beam and column was 30 mm, while the finer mesh size of 20 mm was used for the angle. The finer meshes were needed in the regions of holes in all elements to minimize the effect of the stress concentrations. Thus, a very fine mesh size of 4 mm was used for the bolts and the areas around the holes, resulting in a total number of elements and nodes of the model are 13,208 and 20,800, respectively. The mesh sensitivity analysis revealed that decreasing the mesh size further beyond what is shown in Figure 5 had a minimal impact on the numerical results. However, a significant computational effort and an extensive computer processing time were required to perform the analysis. Pin supports at both ends of columns were modeled to simulate the boundary conditions of the experimental study. In addition, reference points were assigned to calculate the reaction forces easily, rotations, and deformations at different cycles of the analysis.

### 3.2. Material Models

**Figure 6:**
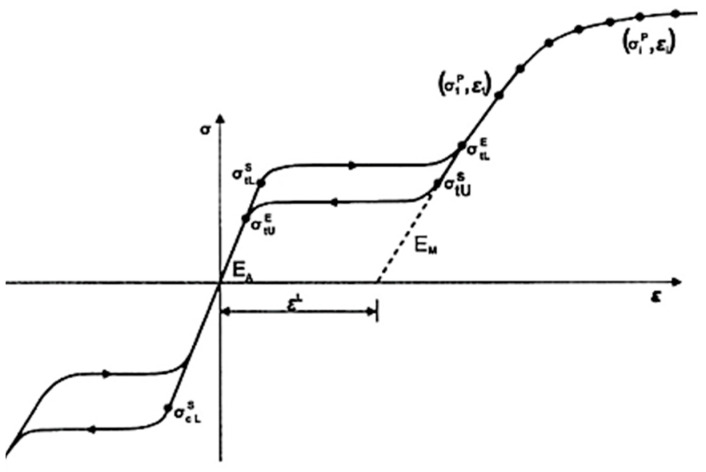
Performance of superelastic material under uniaxial tension (ABAQUS Manual) [42].

The properties of steel elements used in the experimental reference [33] were also implemented in the numerical model. Table 1 provides a summary of the mechanical properties of steel. The SMA elements were defined using the newly built-in superelasticity model in ABAQUS version 2019 and newer [42], as shown in Figure 6. The definition of superelasticity in ABAQUS is based on the constitutive relationships proposed by Auricchio and Taylor work [49,50]. It has been shown that the superelasticity model implemented in this study was capable of simulating the force-deformation relationship of SMA. Several researchers have successfully validated the outcomes of this model in their numerical studies including Higazey et al. [5], Shrestha and Hao [51], and Fugazza [52]. The SMA characteristics used in the FE model were based on the experimental cyclic load test carried out by Ghassemieh et al. [53] for NiTi, and by Omori et al. [54] for FeMnAlNi. Table 2 summarizes the mechanical properties of SMA used in this study.

## 4. Validation of the FE Model

**Figure 7:**
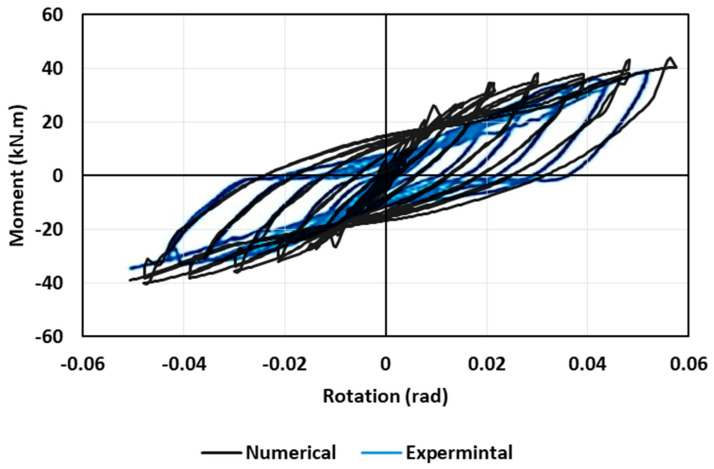
Hysteretic response of experimental by Serrano-López et al. [33] and numerical of the current study. Reprinted from [33], with permission from Elsevier.

**Figure 8:**
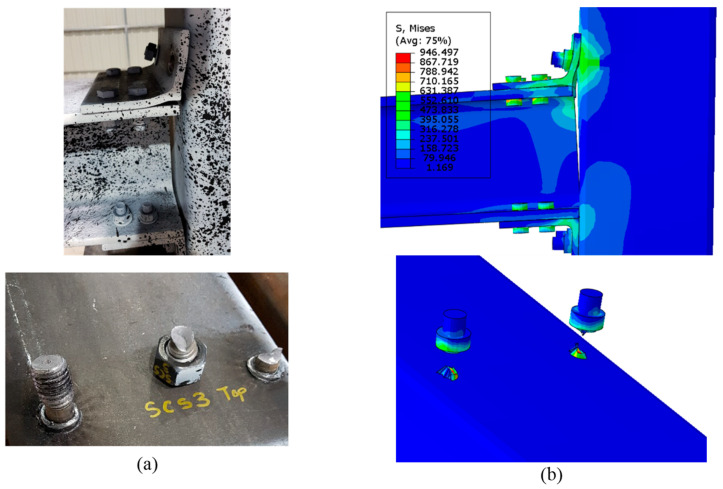
Tensile failure in the welded bolts (**a**) Serrano-López et al. Reprinted from [33], with permission from Elsevier. (**b**) Current study.

The first phase of this investigation was validation of the numerical modelling with the experimental results reported by Serrano-López et al. [33]. The reversed cyclic loading was numerically performed in ABAQUS in a similar manner to the loading protocol conducted in the experimental study, which was in accordance with FEMA-461 [43]. Figure 7 shows a comparison between the numerical versus experimental hysteresis loops. The numerical results seem to compare fairly well with the experimental test results, with a variation of 3% to 5%. Table 3 illustrates the variations in the moment capacity and energy dissipation between the experimental and numerical studies. Moreover, the beam-column connection exhibited a tensile failure mode in the welded bolts of the specimen tested. Figure 8 shows that the FE model was capable of simulating the performance of the beam-column connection and the failure mode was consistent with the failure observed experimentally.

## 5. Numerical Study

**Figure 9:**
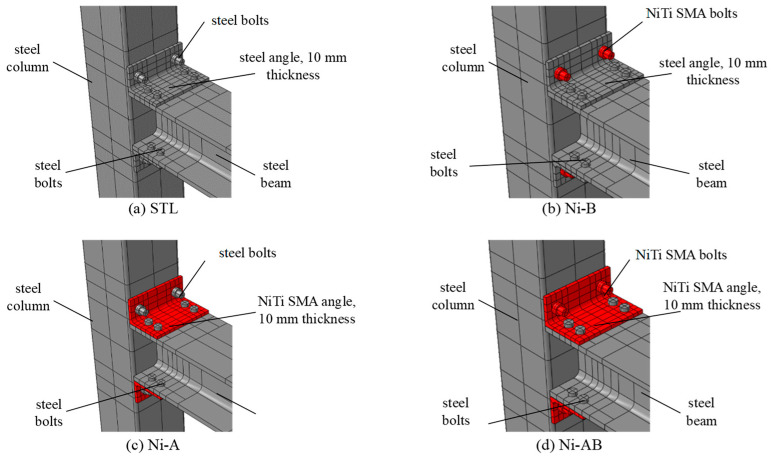
Details of the schemes used in the numerical study.

Following the validation of the FE model with the experimental results of the I-beam-hollow column steel connection [33] in the first phase of this study, the second phase aimed at investigating the cyclic behavior of the connection when the steel angles and bolts are replaced with SMA angles and bolts. Therefore, four innovative approaches were used for studying the effect of the SMA on the cyclic behavior of the connections compared to conventional steel connections: (a) steel bolts and steel angles (b) SMA bolts and steel angles, (c) steel bolts and SMA angles, (d) SMA bolts and SMA angles. The specimens were labeled as X-Y where X represents the alloys (Ni for NiTi) and Y represents the elements replaced with SMA (A, B, and C denote angles, bolts, and both bolts and angles, respectively). The test matrix and schematic drawings of the investigated specimens in the numerical study are presented in Table 4 and Figure 9, respectively.

## 6. Numerical Results

The results of the FE models under the effects of reversed cyclic loadings are summarized in Table 5 and thoroughly discussed in the following sections in terms of the hysteresis response, residual drift, self-centering factor, energy dissipation, moment capacity, initial rotational stiffness, and stiffness degradation.

### 6.1. Hysteretic Response

**Figure 10:**
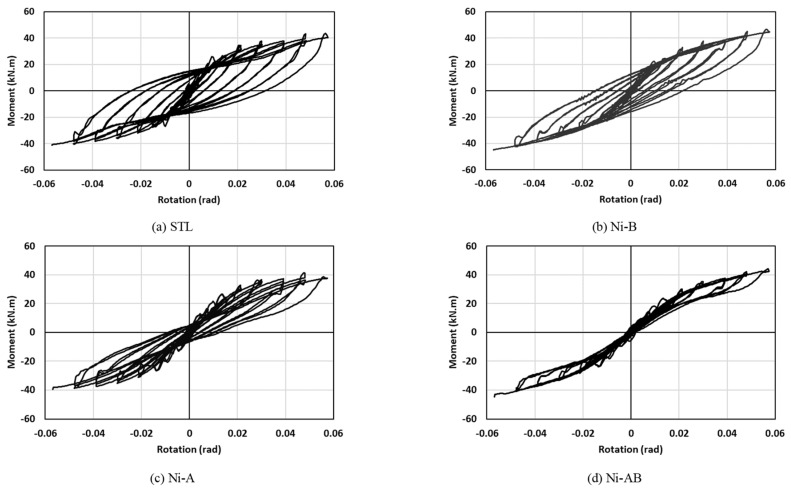
Hysteretic moment-rotation response of the specimens: (**a**) STL (**b**) Ni-B (**c**) Ni-A (**d**) Ni-AB.

The hysteretic moment-rotation curves of all the beam-column connections (STL, Ni-B, Ni-A, and Ni-AB) are presented in Figure 10. The results showed that the use of SMA elements in the beam-column connection in the form of bolts (Ni-B), angles (Ni-A), or a combination of both bolts and angles (Ni-AB) was found to be very effective in reducing the residual drift and enhancing the self-centering capability of the connections, without a noticeable reduction in the moment capacity. The improvement in the residual drift and self-centering was more pronounced when the SMA bolts and angles (Ni-AB) were used compared to the other specimens. The hysteretic moment-rotation response of the Ni-AB exhibited a flag shaped loop and showed the ability of the SMA to recover almost all deformations at higher strain cycles. The variations in the hysteretic moment-rotation curves between the STL and Ni-AB were attributed to the inherent mechanical properties of the SMA. The hysteretic moment-rotation behavior of the Ni-A and Ni-B were influenced by the mechanical characteristics of both steel and SMA, which resulted in behavior falling between the behavior of STL and Ni-AB. From an initial rotational stiffness and energy absorption perspectives, the steel connections (STL) appeared to perform better than the others SMA connections (Ni-B, Ni-A, and Ni-AB). Nevertheless, unlike the steel connection (STL), where the permanent deformations start to occur under early cyclic loading and the behavior is dictated by the energy dissipation to provide enough ductility to the system, the SMA connections are capable of withstanding higher cyclic loads with little to none permanent deformations.

### 6.2. Moment Capacity

**Figure 11:**
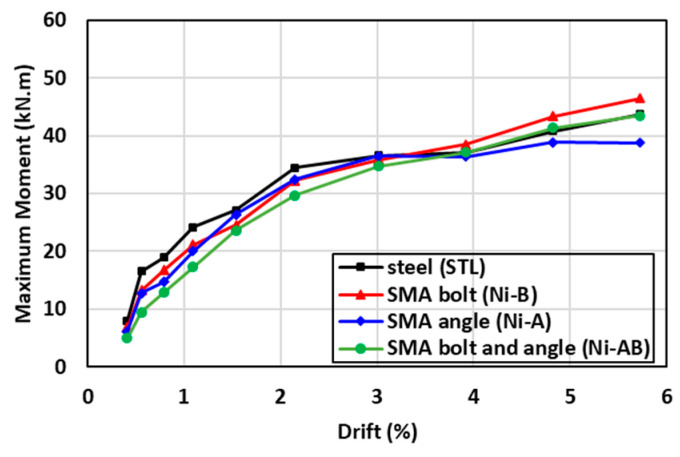
Comparisons of the moment capacity of the specimens tested.

The maximum moment capacity of all specimens versus the corresponding drift ratio is presented in Figure 11. The results showed that the use of SMA in the forms of bolts (Ni-A), angles (Ni-B), or a combination of both angles and bolts (Ni-AB), has an insignificant influence on the moment capacity of the beam-column connections compared to the conventional steel connection (STL). It can be observed that the specimens equipped with SMA elements showed slightly lower values compared to steel specimens up to a drift of 3%. However, at higher drift values, the Ni-A resulted in 6.1% higher value compared to the STL and Ni-AB. On the other hand, using the SMA angle reduced the maximum moment by 5.5% compared to the control specimen. In addition, while the Ni-A and Ni-AB enhanced the moment capacity of the beam-column connection, the moment capacity of the Ni-B was slightly lower than that of the STL. This reduction was attributed to the difference in the stiffness between the steel bolts and SMA angles, which caused some redistribution of stresses at higher loads leading to formation of plastic strain in the bolts and ultimately resulting in a tensile failure in the welded bolts.

**Figure 12:**
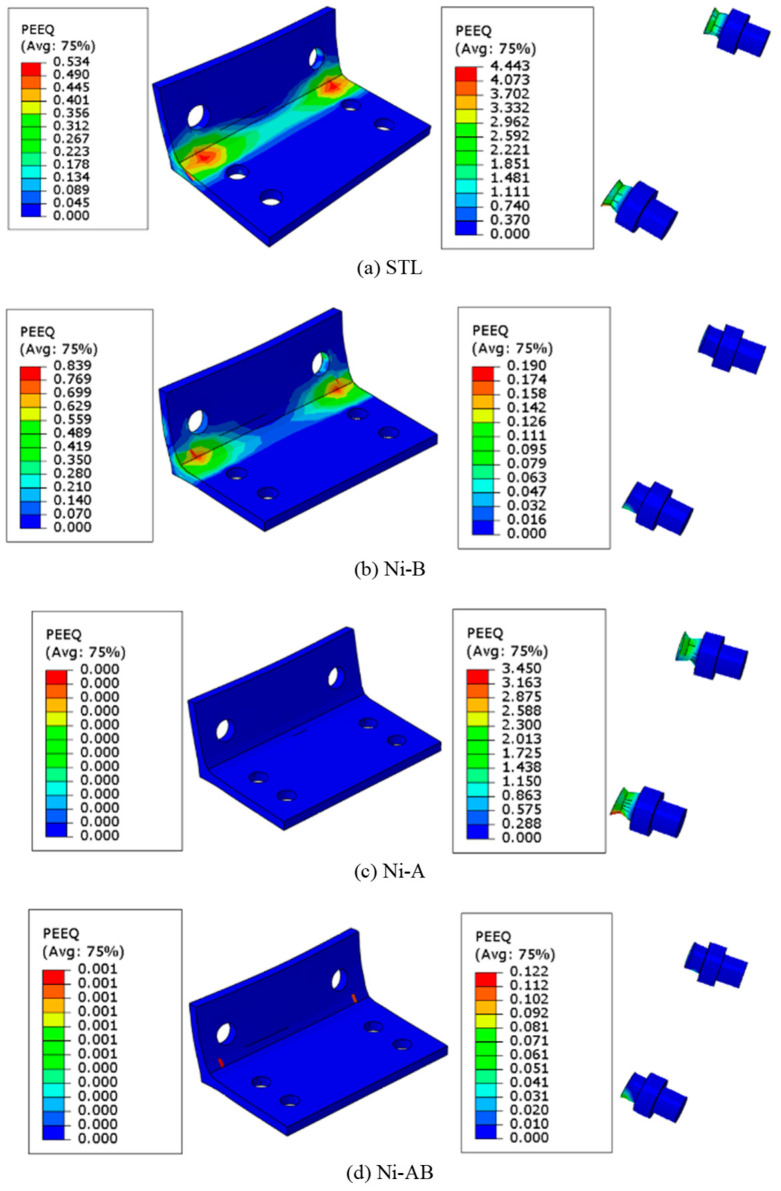
Comparisons of the equivalent plastic strain of the specimens tested.

The failure mode presented in Figure 12 clearly shows that the STL experienced plastic strains in both the bolts and angles of the beam-column connection and the plastic strain recorded in the bolts were very high, 5 times higher than the plastic strain of the angles. This observation was also noticed in the numerical FE models. The numerical results of the FE models were consistent with the experimental failure mode (tensile failure mode in the bolts), as shown in Figure 8. Although the failure mode of the other specimens (Ni-B, Ni-A, and Ni-AB) was also dominated by tensile failure of the bolts, they have exhibited different plastic strain distributions across the bolts and angles. The plastic strains of the Ni-B were severely concentrated in the steel angles at the junction where the two plates form the angle while the SMA bolts experienced slightly lesser plastic strain, roughly 25% of the plastic strains observed in the angles. However, the Ni-A showed quite a different mechanism. The SMA angles had no plastic strain whereas the steel bolts were found to record high plastic strain values. When the steel bolts and angles were replaced by SMA (Ni-AB), the plastic strains were substantially reduced compared to the STL. The SMA angles experienced roughly no plastic strain, while the plastic strain in the bolts was insignificant. The formation of plastic strains does not imply failure in the case of the SMA material. Instead, it implies that the deformations experienced by the angles or the bolts are irrecoverable. The bolts and angles begin to fail when the ultimate stresses exceed the ultimate strength. This explains the tensile failure in the bolts of the Ni-AB, while having very little plastic strains compared to the other cases.

### 6.3. Rotational Stiffness

**Figure 13:**
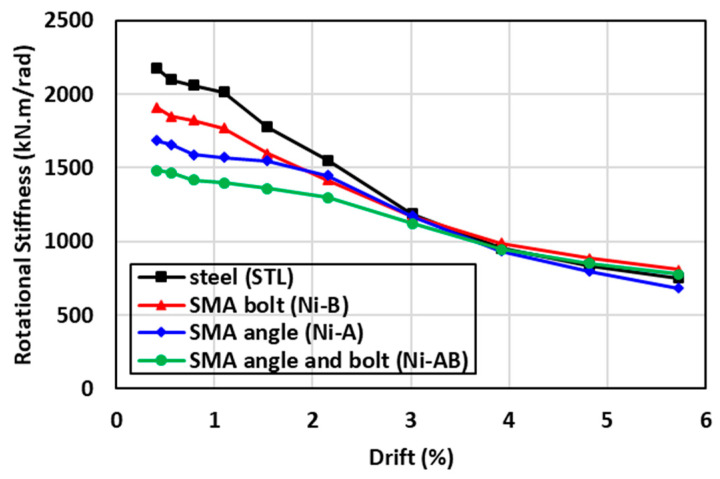
Comparisons of the stiffness degradation of the specimens tested.

The recorded moments and their corresponding drifts at the end of each half cycle were used to calculate the rotational secant stiffness of each specimen [55]. The rotational secant stiffness in each cycle was calculated using a line drawn between the maximum positive drift point in one half of the cycle and the maximum negative drift point in the other half of the cycle. This secant stiffness is intended to provide a qualitative measure of the stiffness degradation of the specimens. The comparison of the rotational stiffness degradation is plotted versus the corresponding drift ratio in Figure 13 for each specimen tested. It can be observed that while the STL had the highest initial rotational stiffness compared to the other specimens, its rotational degradation rate was substantial such that beyond 3% drift the Ni-A, Ni-B, and Ni-AB performed as good as the STL. The results showed that the Ni-B and Ni-A resulted in a lowered initial rotational stiffness by 12% and 23%, respectively. In addition, a further reduction in the initial rotation stiffness by 32% was also noticed in the Ni-AB. The rate of rotational stiffness degradation was the lowest in the Ni-AB, followed by Ni-A and Ni-B. Moreover, the rotational stiffness of the beam-column connections seems to be similar beyond 3% drift, irrespective of the material used in the bolts and/or the angles.

### 6.4. Residual Drift and Self-Centering Factor

**Figure 14:**
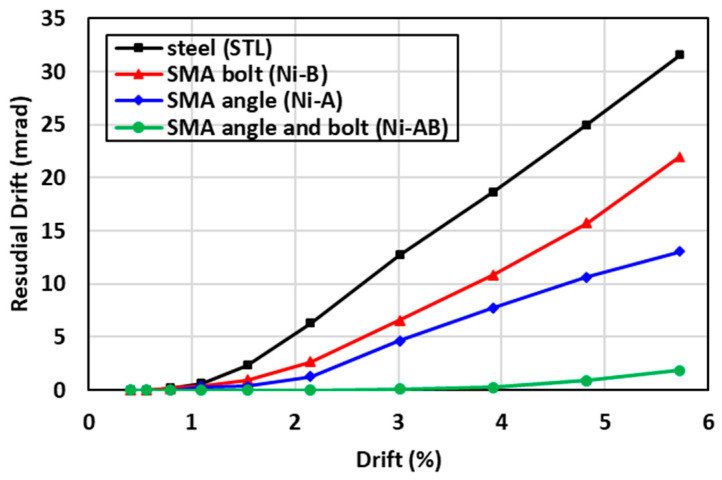
Comparisons of the residual drift of the specimens tested.

Residual drift is defined as the permanent drift of a structure caused by the inelastic deformations due to earthquake excitations [56]. The permanent deformations of structures are a key design criterion that determines the serviceability of the structures and the post-earthquake maintenance cost. Figure 14 presents a comparison between the residual drift values of the beam-column connection. The results indicated that the STL had the highest residual drift whereas the residual drift of the (Ni-AB) specimen was substantially reduced to values close to zero. The use of the SMA in the bolts (Ni-B), in comparison to the STL, resulted in a significant reduction in the residual drift by 30%. Moreover, a further decrease in the in the residual drift of 59% was recorded for the Ni-A, denoting the greater effectiveness of utilizing the SMA angles in controlling the residual drift than the SMA bolts. Interestingly, the use of SMA bolts and angles tremendously minimized the residual drift by almost 94% compared to the STL. The permanent deformations, resulting in the STL (steel beam-column connection), were due to the formation of plastic strains in the vicinity of the steel angles and bolts. Nevertheless, replacing the steel with the SMA decreased these inelastic strains drastically, as shown in Figure 14. The self-centering factors of the specimens investigated were also determined and summarized in Table 1 following Equation (1) as defined by Shajil et al. [57]. The self-centering of the Ni-A and Ni-B were increased to 0.62 and 0.77, respectively compared to 0.45 for STL, as presented in Table 5. These values were drastically increased to 0.97 for Ni-AB.

(1) $$Self\;centering\;factor=\frac{Ultimate\;drift\;-\;\;Residual\;drift}{Ultimate\;drift}$$

### 6.5. Energy Dissipation

**Figure 15:**
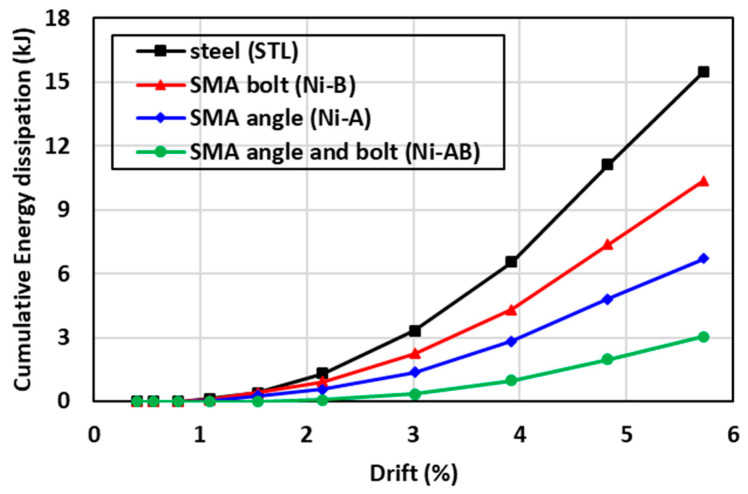
Comparisons of the cumulative energy dissipation of the specimens tested.

**Figure 16:**
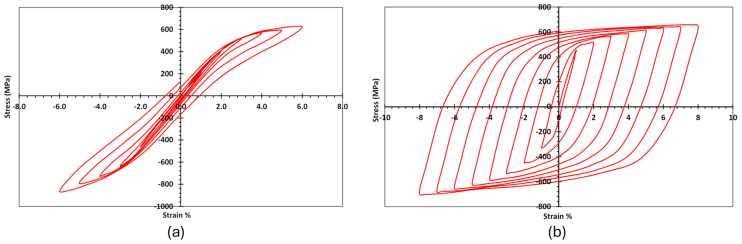
Stress–strain curve of cyclic tension–compression tests: (**a**) Nitinol SMA; (**b**) steel. Reprinted from [59], with permission from American Society of Civil Engineers.

The energy dissipation was calculated for each specimen as the area enclosed by the load-displacement envelopes for each cycle [58]. The cumulative energy dissipation was calculated by summing the values in consecutive cycles up to the end of the testing. The cumulative energy dissipation versus the corresponding drift ratio is presented in Figure 15. The results showed that the STL specimens had the highest cumulative energy dissipation, whereas replacing the steel angles and bolts with SMA (Ni-AB) significantly resulted in the lowest reduction in the cumulative energy dissipation. The reduction in energy dissipation for the SMA connection, compared to the steel connection, could be attributed to differences in the cyclic behavior between the SMA and steel. Nevertheless, the energy dissipation is considered an essential factor in determining the behavior of structure in the post elastic response of the system; the SMA has the distinct advantage of undergoing high strain cycles without causing plastic deformations (4.0–6.0% plastic strain for SMA vs. 0.21% for steel) as shown in Figure 16. The use of SMA bolts (Ni-B) caused a reduction in the energy dissipation by 33% compared to the STL. Additionally, a further reduction in the energy dissipation of about 57% and 80% was also observed when the SMA angles (Ni-A) and both SMA angles and bolts (Ni-AB), respectively, were used.

## 7. Parametric Study

**Figure 17:**
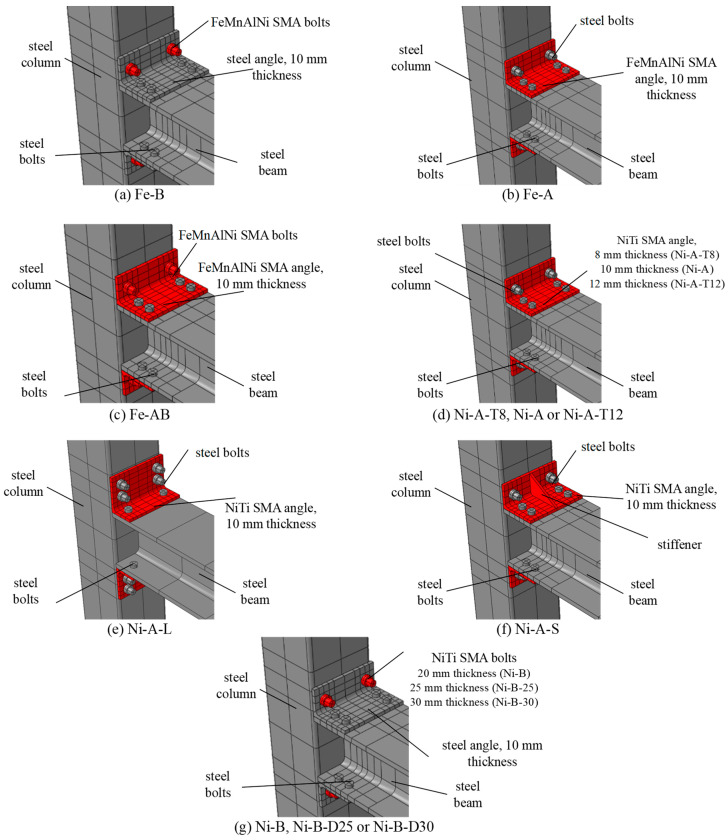
Details of the schemes used in the parametric study.

Following the second phase of this investigation on behavior of the beam-column connections equipped with different SMA elements, the third phase aimed at studying the behavior of the connections for various design parameters. The design parameters investigated include the SMA material type, SMA angle thickness, SMA angle direction, SMA angle stiffener, and SMA bolt diameter. A thorough description of the parameters investigated is summarized in Table 6. The test matrix of the specimens investigated in the parametric study is presented in Table 7. Three structural schemes, used to evaluate the self-centering capability of steel I-beam to square hollow column connections, are (a) SMA bolts, (b) SMA angles, (c) SMA bolts and angles. Each scheme was tested using two SMA materials: (a) NiTi, and (b) FeMnAlNi. The effect of the SMA angle thickness was investigated by considering two angle thicknesses of 8 mm and 12 mm. Additionally, the angle geometry (long leg vs. short leg) was evaluated through the use of a long leg of the SMA angle connected to the column. Furthermore, the addition of stiffeners to the angles was also assessed for the smaller angle thickness of 10 mm. In addition, three SMA bolt dimeters of 20 mm, 25 mm, and 30 mm were considered to assess the contribution of the bolt diameter to the overall behavior of the joint. The specimens were labeled as X-Y-Z, where X represents the alloy material (Ni for NiTi, and Fe for FeMnAlNi) while Y represents the structural steel elements replaced with SMA elements (A for angles, B for bolts, and AB for both angles and bolts). Furthermore, Z represents the effect of the investigated design parameters on behavior of the connections, such as the effect of SMA angle thickness (T8, T10, and T12), the effect of the angle direction (short leg with stiffener denoted as S vs. long leg denoted as L), the effect of the bolt diameter (D20, D25, and D30). Table 6 provides a summary of the design parameters considered in the numerical study, and a detailed test matrix presenting all specimens are listed in Table 7. The schematic drawings of the steel I-beam to square hollow column connections for the design parameters considered in this study are illustrated in Figure 17.

### 7.1. Effect of the SMA Type

**Figure 18:**
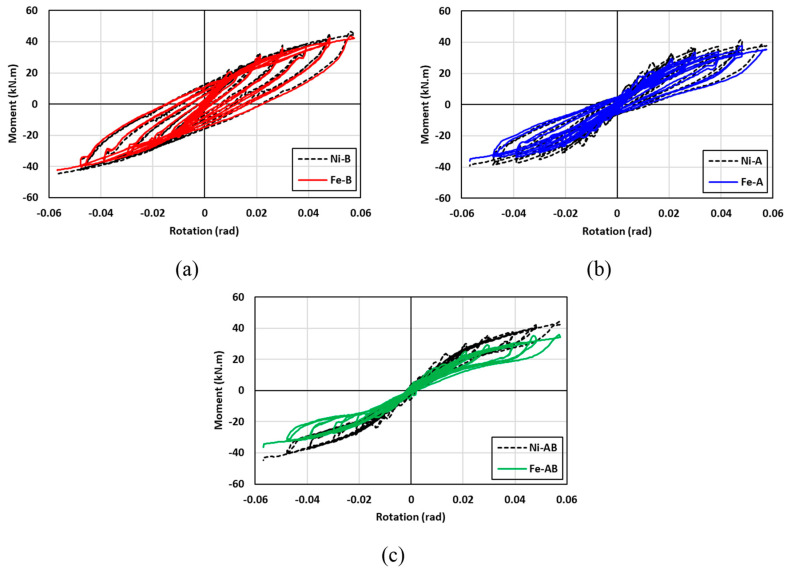
Effect of SMA type on the hysteresis response (**a**) Ni-B vs. Fe-B (**b**) Ni-A vs. Fe-A (**c**) Ni-AB vs. Fe-AB.

**Figure 19:**
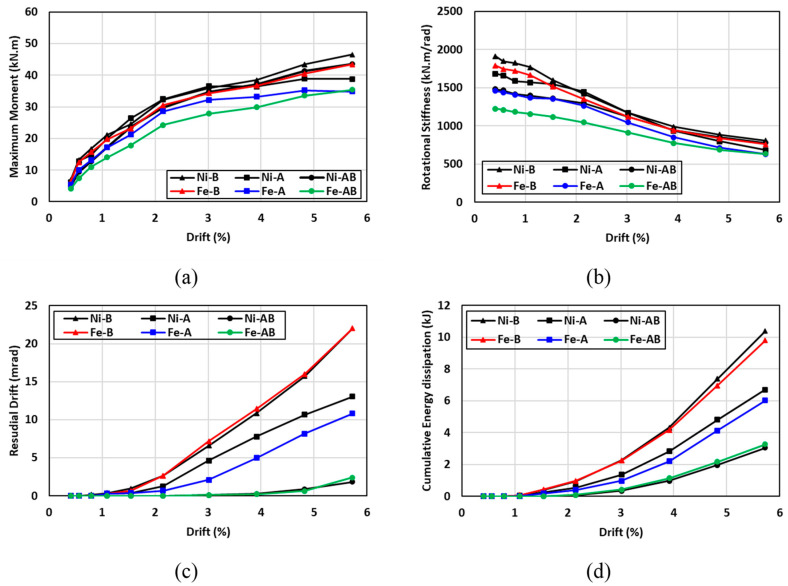
Effect of SMA type on (**a**) maximum moment (**b**) rotational stiffness (**c**) residual drift and (**d**) energy dissipation.

The hysteresis moment-rotation response of the connections fabricated with NiTi and FeMnAlNi SMA are shown in Figure 18. The effect of the SMA material types were investigated for three cases: (a) the angles were only made of SMA (Ni-A vs. Fe-A); (b) the bolts were only made of SMA (Ni-B vs. Fe-B); (c) both angles and bolts were made of SMA (Ni-AB vs. Fe-AB). In general, the hysteresis moment-rotation response seemed to be comparable between the two SMA materials, with a slight increase in the moment capacity for the specimens made of NiTi. The variations between the two hysteresis moment-rotation responses were more noticeable when both angles and bolts were made with SMA materials (Ni-AB vs. Fe-AB), whereas very little to no variations were observed in the hysteresis response when steel was replaced with SMA for the bolts (Ni-B vs. Fe-B). Figure 19 shows the influence of the SMA type on the performance of the connection in terms of the maximum moment, rotational stiffness, residual drift, and energy dissipation. The results indicated that the specimens made with FeMnAlNi SMA exhibited a lowered moment capacity by up to 18% compared to control steel connection and up to 19% compared to specimens with NiTi SMA. This was due to relatively inferior mechanical properties FeMnAlNi SMA compared to the corresponding properties of NiTi SMA and steel. Furthermore, the rotational stiffness of the NiTi specimens showed superior performance than that of the FeMnAlNi specimens at drifts less than 3% and the initial rotational stiffness of the Ni-B, Ni-A and Ni-AB specimens were enhanced by 6%, 13%, and 17% compared to the Fe-B, Fe-A, and Fe-AB specimens, respectively. However, the stiffness degradation rate of the FeMnAlNi and NiTi specimens was very comparable and the deviations between the two materials appeared to diminish beyond 3% drifts. As for the residual drift (self-centering capability), both materials exhibited similar behavior with the exception of the connections utilizing Fe-A and Ni-A mainly in the angles. The variations in residual drift between the Fe-A and Ni-A specimens ranged from 0% to 7%. It is worth mentioning that the use of SMA in both angles and bolts significantly reduced the residual drift to values less than 2% even at higher cycles. The cumulative energy dissipation showed a great similarity of the behavior between the two SMA materials. The highest cumulative energy dissipation was observed for the connections utilizing SMA in bolts only, whereas the use of SMA in both angles and bolts resulted in the lowest cumulative energy dissipation. The difference in cumulative energy dissipation between the FeMnAlNi and NiTi varied between 1.5% and 4.4%. It can be concluded that Fe-based SMA could be a good alternative of NiTi in self-centering applications. 

### 7.2. Effect of SMA Angle Thickness

**Figure 20:**
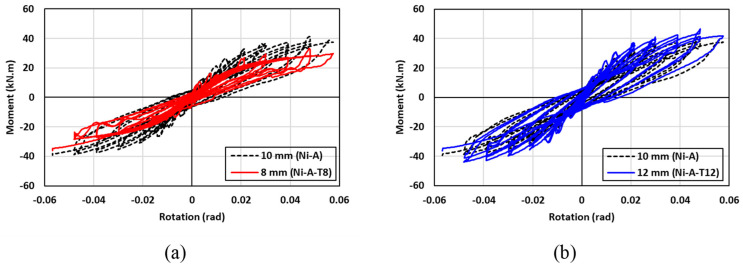
Effect of SMA angle thickness on the hysteresis response (**a**) 10 mm vs. 8 mm (**b**) 10 mm vs. 12 mm.

**Figure 21:**
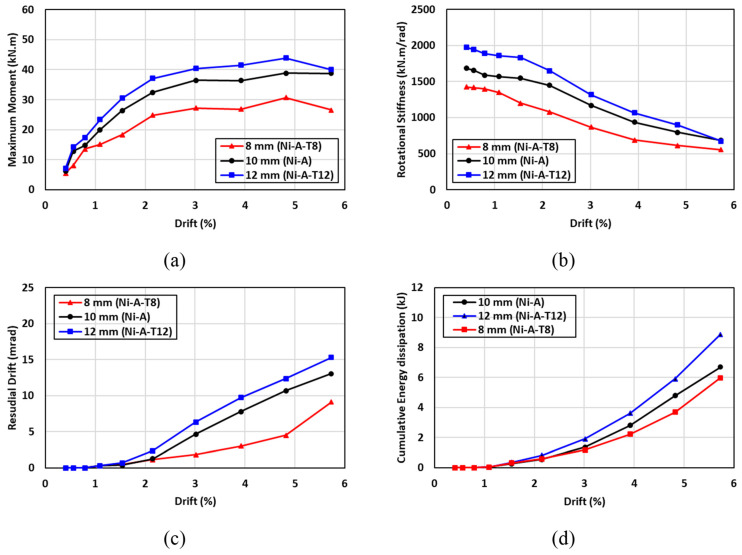
Effect of the SMA angle thickness on (**a**) maximum moment (**b**) rotational stiffness (**c**) residual drift and (**d**) energy dissipation.

The hysteresis moment-rotation response of the connections with three various SMA plate thicknesses is shown in Figure 20. To investigate the effect of the SMA plate thickness on behavior of the beam-column connection, three plate thicknesses of 8 mm, 10 mm, and 12 mm were considered. In general, the hysteresis moment-rotation response seemed to be enhanced as the plate thickness was increased as shown in Figure 20. Figure 21 shows the results of the moment capacity, rotational stiffness, residual drift, and cumulative energy dissipation for the three plate thicknesses. It is observed that increasing the thickness of SMA angle from 8 mm to 12 mm was found to be effective in enhancing the moment capacity and initial rotational stiffness of the connection by up to 40% and 38%, respectively. It should be highlighted that the enhancement in the moment capacity due to the increase in the thickness was more pronounced than the enhancement in the rotational capacity. However, increasing the thickness of SMA angle from 8 mm to 10 mm increased the residual drift by 30%. The cumulative energy dissipation seemed to be slightly influenced by the increase in the plate thickness compared to the other design parameters, especially at low drifts. At higher drifts, the cumulative energy dissipation of the 12 mm plate thickness showed better performance than that of the 8 mm plate thickness by about 30%. It can be concluded that a bigger angle thickness improves the moment capacity and rotational stiffness of the connections. However, the residual drift (self-centering capability) is negatively influenced by the bigger thickness angel. Thus, designers need to balance the strength design requirements against the serviceability limit state.

### 7.3. Effect of SMA Angle Stiffener

**Figure 22:**
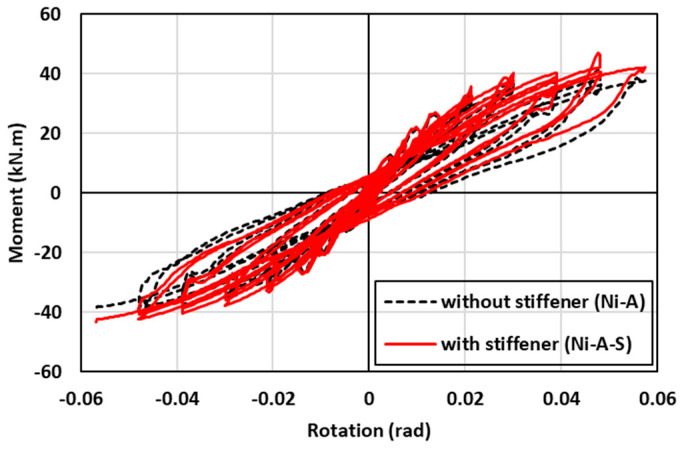
Effect of SMA angle stiffener on the hysteresis response.

**Figure 23:**
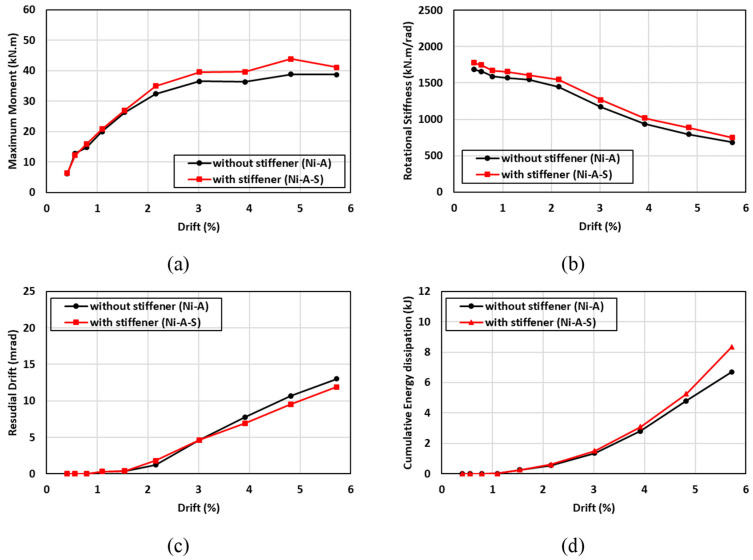
Effect of SMA angle stiffener on (**a**) maximum moment (**b**) rotational stiffness (**c**) residual drift and (**d**) energy dissipation.

The hysteresis moment-rotation response of the connections with and without angle stiffener is shown in Figure 22. To evaluate the contribution of the angle stiffener to performance of the connection, two schemes of connections (with and without angle stiffener) were investigated. In general, the hysteresis moment-rotation response seemed to be slightly improved with the addition of angle stiffener. Figure 23 shows the results of the moment capacity, rotational stiffness, residual drift, and cumulative energy dissipation for the two cases. While the moment capacity of the two systems was almost the same up to 2% drift, the use of an angle stiffener enhanced the moment capacity by 13.5% at higher drifts. Furthermore, a minor improvement of about 4.3% in the rotational stiffness was observed for the connection with angle stiffener. However, comparable residual drift and cumulative energy dissipation responses were noticed between the two connections. It can be concluded that the use of angle stiffener had insignificant contribution to the behavior of the SMA connection given the labor-intensive and time and energy-consuming efforts to fabricate stiffeners in practice.

### 7.4. Effect of the SMA Angle Orientation

**Figure 24:**
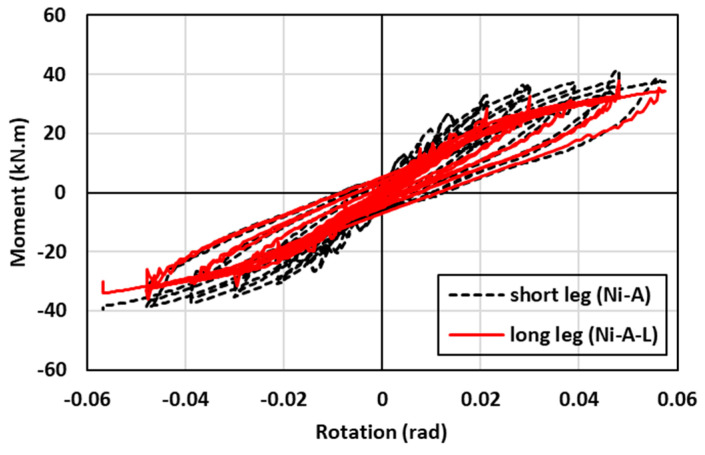
Effect of SMA angle direction on the hysteresis response.

**Figure 25:**
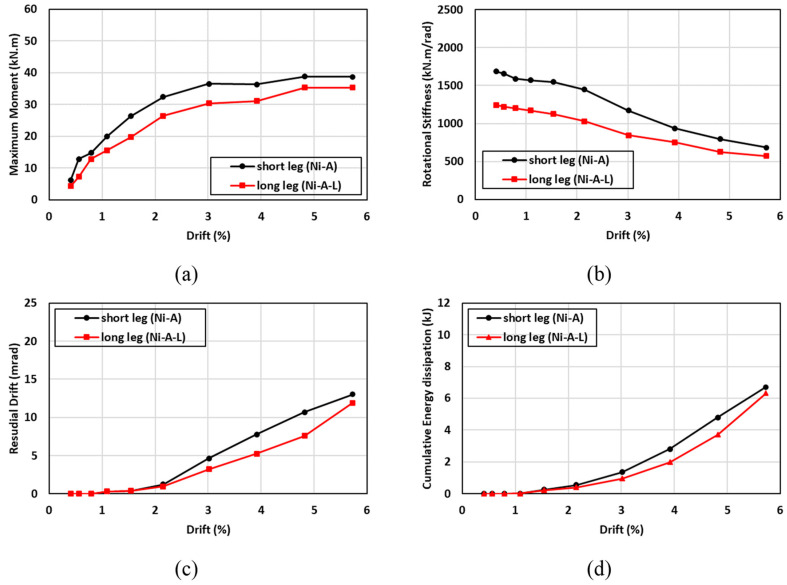
Effect of SMA angle direction on (**a**) maximum moment (**b**) rotational stiffness (**c**) residual drift and (**d**) energy dissipation.

The hysteresis moment-rotation response of the connections with different angle orientations is shown in Figure 24. Two angle orientations (long leg vs. short leg) were considered to determine the effect of the angle orientation on the performance of the connection. The first angle orientation (Ni-A) consisted of two legs in which the short leg of the angle (80 mm) was connected to the column and the long leg of the angle (120 mm) was connected to the beam, as shown in Figure 9c. In contrast, the second angle orientation (Ni-A-L) was fabricated such that the long leg of the angle (120 mm) was connected to the column and the short leg of the angle (80 mm) was connected to the beam, as shown in Figure 17e. In general, the hysteresis moment-rotation response appeared to be improved with the short leg compared to the long leg. Figure 25 shows the results of the moment capacity, rotational stiffness, residual drift, and cumulative energy dissipation for both cases. The initial rotational stiffness, moment capacity, and cumulative energy dissipation of (Ni-A) specimen were 26%, 8%, and 6% higher than (N-A-L) specimen, respectively. Nevertheless, the (N-A-L) specimen slightly performed better than the (Ni-A) specimen. It can be observed that the variations of residual drift between the long and short legs did not exceed 6% and the discrepancies between the two curves were minor. Generally, the advantages of connecting the short leg to the column outperformed the connection of long leg to the same column.

### 7.5. Effect of the SMA Bolt Diameter

**Figure 26:**
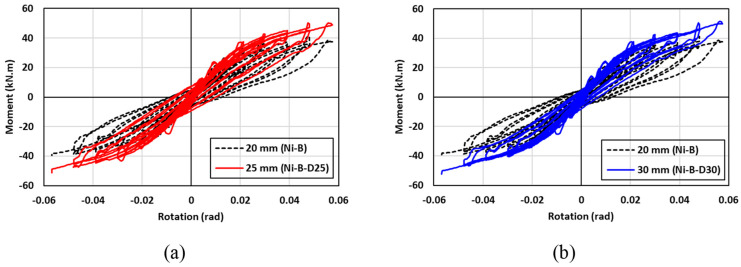
Effect of SMA bolt diameter on the hysteresis response (**a**) 20 mm vs. 25 mm; (**b**) 20 mm vs. 30 mm.

**Figure 27:**
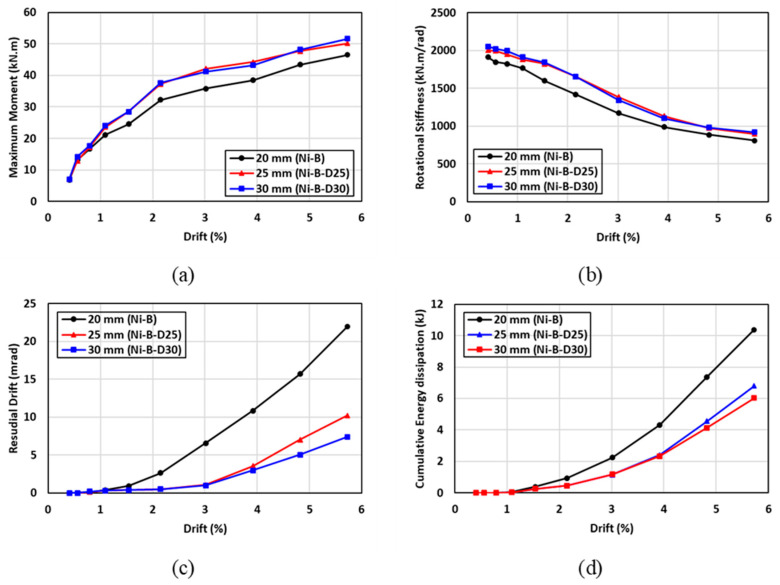
Effect of SMA bolt diameter on (**a**) maximum moment (**b**) rotational stiffness (**c**) residual drift and (**d**) energy dissipation.

The hysteresis moment-rotation response of the connections with different bolt diameters is shown in Figure 26. Three bolts diameters of 20 mm, 25 mm, and 30 mm were considered to determine the influence of the bolt diameter on the performance of the connection. In general, the hysteresis moment-rotation response appeared to be improved with increasing the bolt diameter. Figure 27 shows the results of the moment capacity, rotational stiffness, residual drift, and cumulative energy dissipation for three bolt diameters. These results indicated that increasing the bolt diameter from 20 mm to 25 mm, improved the moment capacity and rotational stiffness by 11% and 7%, respectively, whereas similar behavior was observed for the connections with 25-mm and 30-mm bolt diameters. Nevertheless, the connections with 20-mm and 25-mm bolt diameters showed insignificant variations in the residual drifts and cumulative energy dissipation, whereas the 20-mm bolt diameter resulted in considerable residual drift and cumulative energy dissipation by 40% and 42%, respectively compared to the other diameters. It can be concluded that there is an optimum bolt diameter to achieve the best performance, which was 25 mm in this case, beyond which insignificant improvement can be attained.

## 8. Conclusions

The cyclic performance of steel I beam-hollow column connection using (SMA) bolts and seat angles was numerically investigated through finite element (FE) analysis in ABAQUS software. This is followed by validating the numerical model using experimental data existing in the literature. The following conclusions can be drawn from this investigation:

- The developed 3D finite element model was capable of simulating successfully the cyclic performance of the steel I beam-hollow column connection. The numerical results compared fairly well to those obtained from the experimental test results.
- The connection equipped with a smart actuating system, consisting of the SMA bolts and SMA angles, reduced the residual drift by up to 94%, and doubled the self-centering capability, compared to conventional steel connections.
- The connections equipped with SMA elements exhibited a slower reduction in stiffness degradation and initial rotational stiffness, compared to conventional steel connections.
- The numerical results showed that utilizing SMA angles and SMA bolts provided a significant enhancement in self-centering, and caused a reduction in the other design parameters. However, the use of SMA bolts and steel angles resulted in enhanced energy dissipation, moment capacity, and initial rotational stiffness, and reduced self-centering. The behavior of the I beam-hollow column connection equipped with SMA angles and steel bolts was in between the performance of the previous two cases.
- Among the types of smart materials investigated, Fe-based SMA members could be a good alternative to NiTi based SMA members for improving the self-centering capability and reducing the residual drifts of conventional steel connections.
- Among the parameters investigated, increasing the SMA bolt diameter, and SMA plate thickness, caused a significant increase in self-centering capability of steel connections, without compromising their moment capacity.
- Generally, the advantages of connecting the short leg of SMA angle to hollow section steel column outperformed the connection of long leg of SMA angle to the same column.
- The numerical results of the parametric study reported herein, could serve as a valuable reference for optimizing the performance of the steel I beam-hollow column connections equipped with smart actuating systems, subjected to reversed cyclic loading.
- Additionally, minimizing the permanent deformations through the use of SMAs, would reduce the maintenance requirements of structures built in active seismic zones, and thus boost their applications in various types of structures.

## Figures and Tables

**Table 1:** Mechanical properties of steel elements [33].

Element		Yield Strength (MPa)	Ultimate Strength (MPa)	Elastic Modulus (GPa)
Column (SHS200 × 10)		417.0	520.1	215.3
Beam (HEB 200)	Flange	325.2	492.7	195.7
	Web	334.9	482.0	192.3
Angle (L120 × 80 × 10)	Long leg	316.6	468.8	176.4
	Short leg	322.5	469.6	201.5

**Table 2:** Mechanical properties of SMA angles used in ABAQUS.

Alloy	Start of Transformation (Loading)	End of Transformation (Loading)	Start of Transformation (Unloading)	End of Transformation (Unloading)	Young’s Modulus	Transformation Strain
	$\sigma_{tL}^{s}$ (MPa)	$\sigma_{tL}^{E}$ (MPa)	$\sigma_{tU}^{s}$ (MPa)	$\sigma_{tU}^{E}$ (MPa)	E_A_ (GPa)	$\varepsilon^{L}$
NiTi [53]	435	535	335	170	68	0.08
FeMnAlNi [54]	320	443	211	122	98	0.06

**Table 3:** Comparison between the numerical results of the current study and the experimental values of the tested connection.

	Experimental Study [33]	Current Study	Variation, %
Maximum Moment (kN-m)	41.67	43.77	5.04
Energy dissipation (kJ)	15.02	15.50	3.21

**Table 4:** Details of the specimens used in the numerical study.

Sample ID	Material of Angles	Material of Column’s Bolts	Material of Beam’s Bolts	Material of the Beam	Material of the Column
STL	Steel	Steel	Steel	Steel	Steel
Ni-B	Steel	NiTi SMA	Steel	Steel	Steel
Ni-A	NiTi SMA	Steel	Steel	Steel	Steel
Ni-AB	NiTi SMA	NiTi SMA	Steel	Steel	Steel

**Table 5:** The numerical results of the FE model of the beam-column connections.

Sample ID	Residual Drift (mrad)	Self-Centering Factor	Maximum Moment (kN-m)	Initial Rotational Stiffness (kN-m/rad)	Total Energy Dissipation (kJ)	Failure Mode
STL	31.6	0.45	43.8	2177.4	15.50	Bolt failure in tension
Ni-B	22.0	0.62	46.5	1911.6	10.39	Bolt failure in tension
Ni-A	13.0	0.77	41.4	1683.5	6.70	Bolt failure in tension
Ni-AB	1.8	0.97	44.2	1481.4	3.03	Bolt failure in tension

**Table 6:** Summary of investigated parameters used in the numerical investigation.

#	Parameter	Variables	Related Specimens
1	SMA type	(a) NiTi	Ni-B, Ni-A and Ni-AB
		(b) FeMnAlNi	Fe-B, Fe-A and Fe-AB
2	Thickness of SMA angle	(a) 8 mm	Ni-A-T8
		(b) 10 mm	Ni-A
		(c) 12 mm	Ni-A-T12
3	Direction of SMA angle	(a) short leg connected to the column	Ni-A
		(b) long leg connected to the column	Ni-A-L
4	Stiffener of SMA angle	(a) without stiffener	Ni-A
		(b) with stiffener	Ni-A-S
5	Diameter of SMA bolt	(a) 20 mm	Ni-B
		(b) 25 mm	Ni-B-D25
		(c) 30 mm	Ni-B-D30

**Table 7:** Test matrix of the specimens used in the numerical investigation.

#	Sample ID	Angle Material	Angle Thickness	Angle Direction (Connected Leg to the Column)	Material Of Column’s Bolt	Diameter of Column’s Bolt
1	STL	Steel	10 mm	Short leg	Steel	20 mm
2	Ni-B	Steel	10 mm	Short leg	NiTi	20 mm
3	Ni-A	NiTi	10 mm	Short leg	Steel	20 mm
4	Ni-AB	NiTi	10 mm	Short leg	NiTi	20 mm
5	Fe-B	Steel	10 mm	Short leg	FeMnAlNi	20 mm
6	Fe-A	FeMnAlNi	10 mm	Short leg	Steel	20 mm
7	Fe-AB	FeMnAlNi	10 mm	Short leg	FeMnAlNi	20 mm
8	Ni-A-T8	NiTi	8 mm	Short leg	Steel	20 mm
9	Ni-A-T12	NiTi	12 mm	Short leg	Steel	20 mm
10	Ni-A-S *	NiTi	10 mm	Short leg	Steel	20 mm
11	Ni-A-L	NiTi	10 mm	Long leg	Steel	20 mm
12	Ni-B-D25	Steel	10 mm	Short leg	NiTi	25 mm
13	Ni-B-D30	Steel	10 mm	Short leg	NiTi	30 mm

* Angle stiffener was used in this specimen.

## Data Availability

The datasets generated and/or analyzed during the current study are available from the corresponding author upon reasonable request.
